# The triterpenoid sapogenin (2α-OH-Protopanoxadiol) ameliorates metabolic syndrome via the intestinal FXR/GLP-1 axis through gut microbiota remodelling

**DOI:** 10.1038/s41419-020-02974-0

**Published:** 2020-09-17

**Authors:** Zhifu Xie, Haowen Jiang, Wei Liu, Xinwen Zhang, Dakai Chen, Shuimei Sun, Chendong Zhou, Jia Liu, Sheng Bao, Xiachang Wang, Yinan Zhang, Jia Li, Lihong Hu, Jingya Li

**Affiliations:** 1 Shanghai Institute of Materia Medica, Chinese Academy of Sciences, Shanghai, 201203 P.R. China; 2 University of Chinese Academy of Sciences, Beijing, 100864 P.R. China; 3 Jiangsu Key Laboratory for Functional Substance of Chinese Medicine, Nanjing University of Chinese Medicine, Nanjing, 210023 P.R. China

**Keywords:** Diabetes, Drug development

## Abstract

Gypenosides, extracts of *Gynostemma yixingense*, have been traditionally prescribed to improve metabolic syndrome in Asian folk and local traditional medicine hospitals. However, the mechanism of its action remains unclarified. In this work, our results indicated that chronic administration of 2α-OH-protopanoxadiol (GP2), a metabolite of gypenosides in vivo, protected mice from high-fat diet-induced obesity and improved glucose tolerance by improving intestinal L-cell function. Mechanistically, GP2 treatment inhibited the enzymatic activity of bile salt hydrolase and modulated the proportions of the gut microbiota, which led to an increase in the accumulation of tauro-β-muricholic acid (TβMCA) in the intestine. TβMCA induced GLP-1 production and secretion by reducing the transcriptional activity of nuclear receptor farnesoid X receptor (FXR). Transplantation of GP2-remodelled fecal microbiota into antibiotic-treated mice also increased the intestinal TβMCA content and improved intestinal L-cell function. These findings demonstrate that GP2 ameliorates metabolic syndrome at least partly through the intestinal FXR/GLP-1 axis via gut microbiota remodelling and also suggest that GP2 may serve as a promising oral therapeutic agent for metabolic syndrome.

## Introduction

Metabolic syndrome, the chronic disease caused by a cluster of nutritive risk factors, has become a major public-health challenges worldwide^1,2^. Glucagon-like peptide-1 (GLP-1), a potent anti-hyperglycaemic incretin hormone, is produced by and secreted from intestinal L cells and subsequently potentiates pancreatic β-cell glucose-stimulated insulin secretion after food ingestion^3^. Recently, evidence has suggested that the rate of L-cell dysfunction is accelerated owing to intestinal lipotoxicity and oxidative stress in obese patients^4,5^. Therefore, strategies to improve intestinal L-cell function may have novel therapeutic potential for metabolic syndrome.

Bile acids (BAs), which share a carbon skeleton with steroids, are synthesised from cholesterol in the liver and secreted into intestine to facilitate the absorption of dietary lipids and lipid-soluble vitamins^6–8^. Over 95% of bile acids in the intestine are resorbed, the remaining 5% pass into colon where they are modified into secondary bile acids^9^. Glycine- or taurine-conjugated primary BAs are deconjugated rapidly by bile salt hydrolase (BSH) in the intestine, which is widely expressed by the gut microbiota, especially the genera *Bacteroides*, *Lactobacillus* and *Clostridium*^10,11^. BAs have recently emerged as likely regulators of metabolic sensors that modulate energy harvest and expenditure^12,13^, oral of BSH inhibitor improves metabolic disorders by reducing the de-conjugation of BAs and FXR inhibition^14^.

FXR, a member of the nuclear receptor superfamily that is highly expressed in the liver and intestine, has a critical role in the control of hepatic bile acid biosynthesis and enterohepatic circulation^15,16^. Conjugated bile acids, such as glycoursodeoxycholic acid in humans and tauro-β-muricholic acid (TβMCA) in mice, have been identified as FXR antagonists^17–19^. Intestinal FXR inhibition regulates glucose and lipid homoeostasis partly through the gut–liver axis and gut–adipose axis^20–22^. Recent studies indicated FXR inhibition in L cells stimulates GLP-1 production and glucose-induced GLP-1 secretion^14,21,23,24^. Therefore, de-activation of intestinal FXR may be the potential strategy for metabolic syndrome treatment^14,18,19^.

*Gynostemma yixingense* (*G. yixingense*), a natural plant of the family *Cucurbitaceae*, is used as a functional food in Asian countries^25,26^. Gypenosides, a group of dammarane-type triterpenoid saponins isolated from *G. yixingense*, are the principal bioactive constituents responsible for the observed beneficial effects on glucose and lipid homoeostasis, but the underlying mechanism remains poorly understood^27^. In the present study, we revealed that the triterpenoid sapogenin 2α-OH-protopanoxadiol (GP2), a bioactive metabolite of gypenosides in vivo, acts as a BSH inhibitor and modulates the gut microbiota. Oral administration of GP2 in HFD-fed mice improves metabolic syndrome by modulating the intestinal FXR/GLP-1 axis. Our results suggest that GP2 has therapeutic potential for the treatment of metabolic syndrome.

## Materials and methods

### Metabolic animal experiments

Male C57BL/6 J mice and ICR mice were purchased from Shanghai Model Organisms (Shanghai, China). Male *ob/ob* mice were obtained from Jackson Laboratory. Intestinal FXR knockout (FXR^−/−^) mice were kindly provided by professor Cen Xie at Shanghai Institute of Materia Medica^18^. Animal welfare and experimental procedures were performed in accordance with the current guide of the Animal Ethics Committee of the Shanghai Institute of Materia Medica.

For chronic treatment, sample size estimation was performed before experiments. HFD-fed mice were assigned randomly to various treatment according to body weight and blood glucose levels and received oral administration with or without GP2 (200 mg/kg/d, bid). GP2 was dissolved in a final solution containing 0.5% methylcellulose (155496, MP Biomedicals, CA, USA), 1% dimethyl sulfoxide (DMSO; D4540-500mL, Sigma-Aldrich, Missouri, USA) and 1% Kolliphor EL (C5135-500g, Sigma-Aldrich, Missouri, USA).

### BSH activity analysis

BSH activity was determined based on the enzymatic rate of d5-TCDCA deconjugated to d5-CDCA^19^. In brief, feces were sonicated in cold phosphate-buffered saline (1:10, w/v), then centrifuged at 12,000 × *g* for 15 min. The fecal protein solution containing BSH was isolated. A total of 100 μL of assay solution, including 50 μM TCDCA-d5 and 0.1 mg/mL fecal protein, was incubated in 3 mM sodium acetate buffer (pH 5.2) at 37 °C for 20 min. The reaction was quickly stopped with dry ice, and 100 μL of reaction mix was added to an equal volume of methanol and mixed gently. After centrifugation at 12,000 × *g* for 20 min, 20 μL of supernatant was mixed with 20 μL water for analysis.

### 16 S rRNA gene sequencing of the gut microbiota

Fresh feces were collected, frozen immediately and stored at −80 °C before 16 S rRNA sequencing. Bacterial DNA was extracted using a DNA kit (Omega Bio-tek, USA) and amplified with barcoded universal bacterial primers targeting the variable V3–V4 region of the 16 S rRNA gene. The PCR products were extracted from 2.0% agarose gels, purified with the AxyPrep DNA Gel Extraction Kit (Axygen Biosciences, Union City, USA) and quantified by the QuantiFluor™-ST (Promega, Wisconsin, USA). The fecal bacterial DNA was added to Illumina adaptors by ligation (TruSeq DNA LT Sample Prep Kit, Illumina), and the adaptor-ligated DNA fragments were further amplified on an Illumina MiSeq platform for sequencing according to the standard protocols. Operational taxonomic units were clustered with 97% similarity by UCLUST. Principal coordinates analysis (PCoA) and Sobs index analysis were performed with the vegan package for R language^28^.

### Cell culture and in vitro GLP-1 measurements

STC-1 cells (ATCC® CRL-3254™, RRID: CVCL_J405) were cultured and maintained in high glucose-Dulbecco’s Modified Eagle Medium containing 15% (v/v) fetal bovine serum. STC-1 cells were seeded at a density of 1 × 10^5^ cells/ml in a 0.5% matrix gel coated 24-well plates for 24 h. Afterward, the cells were treated with TβMCA or DMSO containing 0.1% fatty acid free bovine serum albumin and 0.1% Dpp-4 inhibitor for 24 h. At the end of this period, the medium was transferred for GLP-1 measurement according to the manufacturer’s instructions (62GLPPEG, Cisbio, France).

### StatisticsPad Prism (GraphPad Software, La Jolla,

All data are presented as the mean ± s.e.m. Statistical analyses were performed with Student’s *t* test in GraphPad Prism (GraphPad Software, La Jolla, CA, USA). *P* values < 0.05 were considered statistically significant.

## Results

### GP2 treatment protects mice from metabolic syndrome and promotes energy expenditure upon high-fat diet feeding

**Fig. 1:**
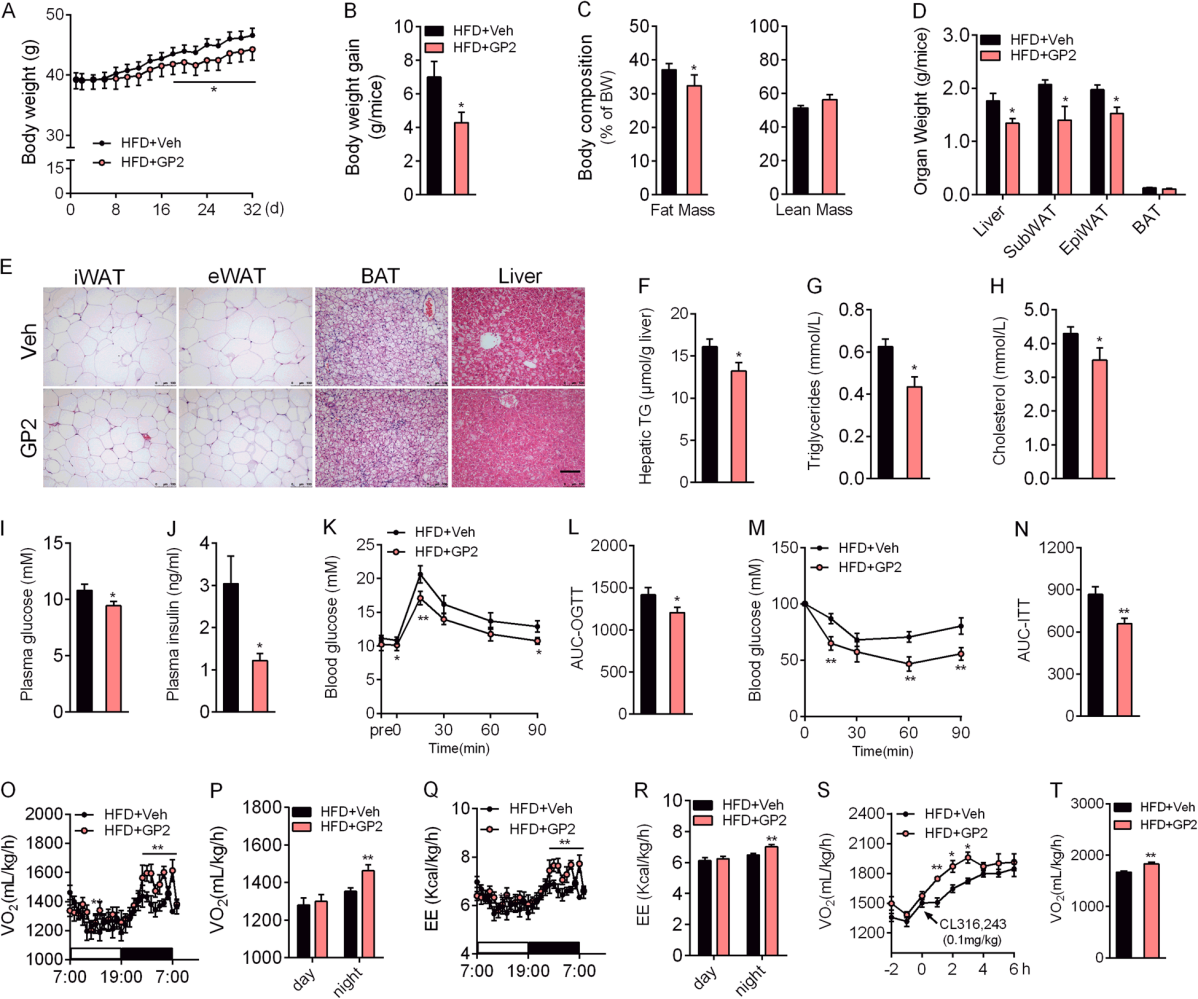
GP2-treated mice are resistant to obesity-associated metabolic disorders. Six-week-old male mice were fed a high-fat diet for 8 weeks, and then treated with or without GP2-200 mg/kg/d for 5 weeks (*n* = 8–10). **a** Body weight changes in 32 days. **b** Body weight gain of mice after 32 days of GP2 treatment. **c** Fat mass and lean mass were assessed by NMR after 4 weeks of GP2 treatment. **d** Organ weight of mice treated with or without GP2. **e** Representative pictures of H&E staining of iWAT, eWAT, BAT and the liver at the end of dietary intervention. Scale bar = 100 μm. **f** Levels of hepatic TG in vehicle-treated and GP2-treated mice. **g**–**j** Mice were fasted for 6 h, and the fasting blood triglyceride (**g**), cholesterol (**h**), glucose (**i**) and insulin (**j**) levels of vehicle-treated mice and GP2-treated mice were measured. **k**, **l** Oral glucose tolerance test (OGTT, 2.0 g/kg) was conducted after three weeks treatment (**k**); the area under curve of glucose level in 90 min of the OGTT (**l**). The results are shown as the mean ± s.e.m., *P < 0.05, **P < 0.01 compared with vehicle.

*G. yixingense* is a traditional medicine for metabolic disorders. We identified a novel sapogenin named GP2, a metabolite of *G. yixingense* in vivo, especially in diabetic mice (Figure S1). To investigate the potential therapeutic effect of GP2 on metabolic syndrome, we gavaged HFD-fed mice with or without GP2 for 5 weeks. Compared with vehicle mice, oral of GP2 significantly attenuated body weight gain and fat mass (Fig. 1a–c), which was associated with reduced adipocyte size in white adipose tissue (Fig. 1d, e). The increase in hepatic lipids induced by high-fat diet was also reduced in GP2-treated mice (Fig. 1e, f), and this finding was consistent with the lower plasma level of the liver enzyme ALT (Figure S2a, S2b).

GP2 treatment decreased plasma triglyceride and cholesterol levels (Fig. 1g, h). Fasting plasma glucose and insulin levels were also much lower than that in vehicle-treated mice (Fig. 1i, j). The oral glucose tolerance test (OGTT) revealed that glucose tolerance capacity of GP2-treated mice was improved (Fig. 1k, l). Insulin tolerance tests (ITT) also demonstrated that the insulin sensitivity of GP2-treated mice was significantly increased (Fig. 1m, n). Accordingly, oral administration of GP2 increased hepatic insulin receptor and AKT phosphorylation (Figure S2c–S2f). These results indicate that gavaged of GP2 improves ameliorates metabolic syndrome in HFD-fed mice.

Metabolic parameter changes in GP2-treated mice were not owing to reduction of caloric intake or triglyceride absorption in the intestine (Figure S2g, S2h), but were the consequence of an increase in energy expenditure (Fig. 1o–r, Figure S2i, S2j). Compared with vehicle mice, GP2 treatment led to more energy expenditure (Fig. 1s, t), but no changes in locomotor activity (Figure S2k, S2l).

To confirm the effect of GP2 on energy expenditure, we subjected mice to cold stress. GP2-treated mice maintained higher body temperature during 4 °C exposure (Figure S3a). In addition, gene expression profiling of BAT confirmed that GP2 treatment increased the levels of brown fat marker genes and several genes involved in β-oxidation. Meanwhile, GP2 treatment slightly increased the protein level of UCP1 (Figure S3b, S3c). These results clearly demonstrate the benefits of GP2 treatment on energy expenditure.

### Chronic GP2 treatment induces pro-glucagon expression and GLP-1 secretion

To identify how GP2 improves glucose homoeostasis, we analysed the absorption and distribution of GP2 after oral administration. Male mice were treated with a single dose of 200 mg/kg GP2, and the concentrations of GP2 in the intestine, plasma and liver tissues were measured. Accumulation of GP2 in the intestine was much higher (62.5 ± 16.47 μg/g) than that in the liver (1.39 ± 1.22 μg/g) or plasma (0.80 ± 0.51 μg/mL) at 2 h post administration (Figure S4a, S4b). Moreover, the absorbed GP2 in the liver was metabolised rapidly in hepatic microsomes (Table S1).

**Fig. 2:**
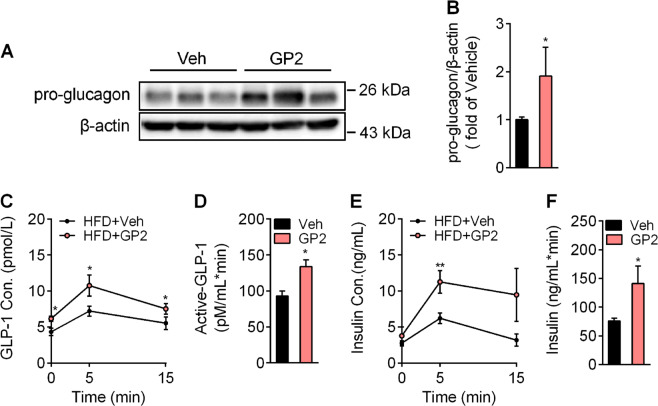
Oral administration of GP2 induces pro-glucagon expression and GLP-1 secretion in the intestine. **a**, **b** Immunoblot (**a**) for pro-glucagon in RIPA lysis extracts from the ilea of mice treated with or without GP2 for 2 weeks and quantification (**b**) (*n* = 5). **c**–**f** Plasma concentrations of active GLP-1 (**c**) and insulin (**e**) in HFD-fed mice in response to oral of 2.0 g/kg glucose with or without GP2 treatment (200 mg/kg/d, bid) for 2 weeks (*n* = 5–6); area under the curve of active GLP-1 level (**d**) and insulin level (**f**) in 15 min after glucose challenge in (**c**) and (**e**). Results are shown as the mean ± s.e.m., *P < 0.05, **P < 0.01 compared with vehicle.

GLP-1 secretion by intestinal L cells improves glucose tolerance. Based on the data from pharmacokinetic analysis of GP2 and its strong distribution in the intestine, we hypothesised that GP2 improves the incretin action of intestinal L cells. To verify the effects of GP2 on GLP-1 action, we detected the intestinal protein levels of pro-glucagon. GP2 treatment induced more pro-glucagon protein expression than vehicle treatment (Fig. 2a). Correspondingly, the plasma concentrations of active GLP-1 and insulin were higher in the GP2-treated mice after glucose challenge (Fig. 2b, c). Meanwhile, the activity of plasma dipeptidyl peptidase-4 (Dpp-4), an adenosine deaminase responsible for the degradation of active GLP-1, was not changed in the GP2-treated mice (Figure S5). These data indicate that improvement of glucose-stimulated GLP-1 secretion results from much more pro-glucagon expression and maturation in endocrine cells after GP2 treatment.

### Oral treatment with GP2 induces intestinal FXR signalling inhibition

**Fig. 3:**
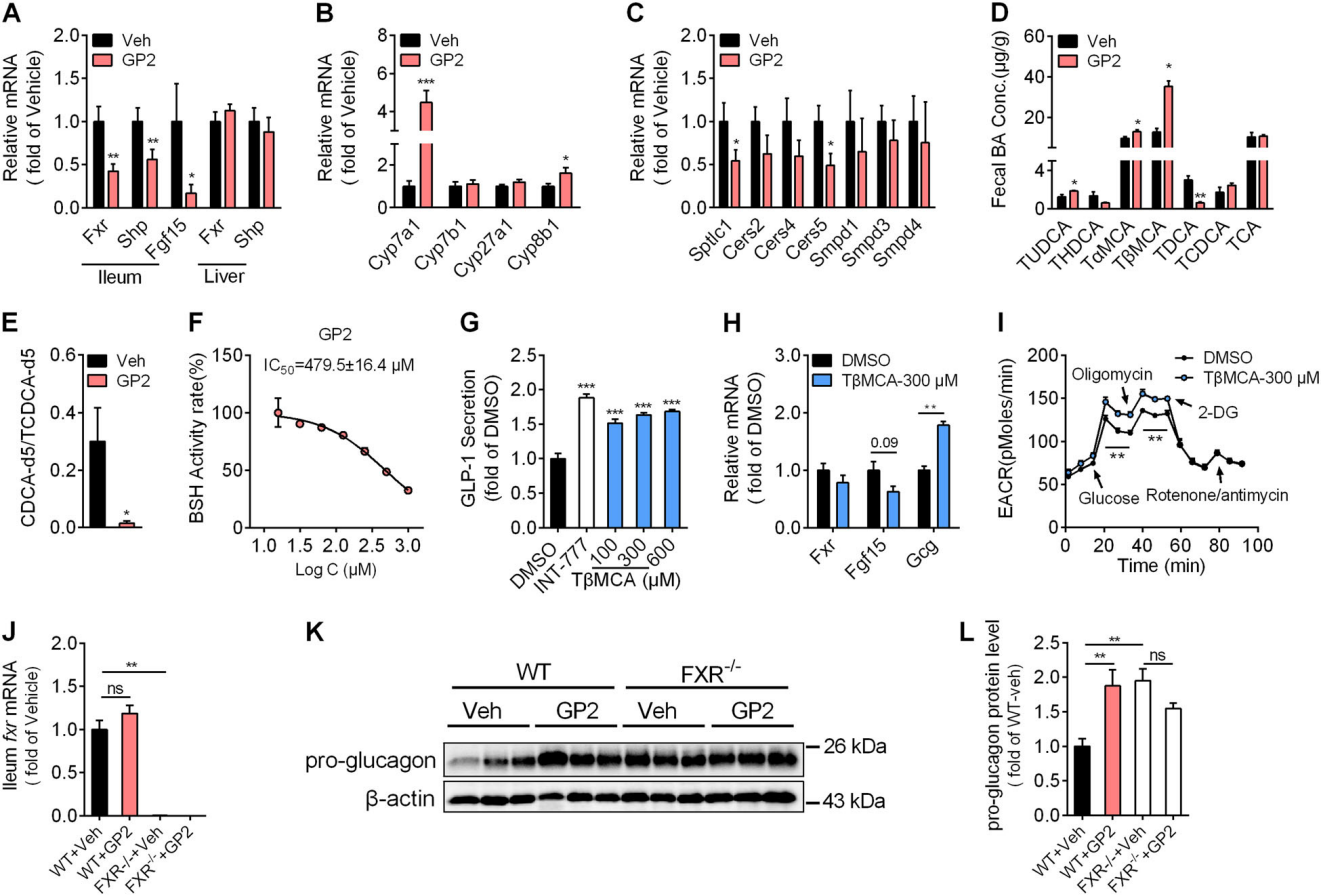
GP2 treatment inhibits the intestinal FXR signalling pathway and improves intestinal L-cell function via accumulation of fecal TβMCA. **a**, **b** mRNA expression of the target genes in the ileum (**a**) and liver (**a**, **b**) (*n* = 7–8). **c** Gene expression of the target genes in the ileum (*n* = 7–8). **d** Individual taurine-conjugated bile acid levels were detected in the feces of HFD-fed mice (*n* = 3). **e** BSH enzymatic activity in the feces of HFD-fed mice treated with or without GP2 (200 mg/kg). The ratio of CDCA-d5 to TCDCA-d5 was used to represent the enzymatic activity of BSH (*n* = 4). **f** BSH enzymatic activity in the feces of HFD-fed mice treated with different concentrations of GP2 in vitro. The ratio of CDCA-d5 to TCDCA-d5 was used to represent the enzymatic activity of BSH (*n* = 3). **g** GLP-1 concentration in the supernatants of STC-1 cells were measured after incubation with DMSO or TβMCA for 24 h, and the TGR5 agonist INT-777 (10 μM) was used as a positive control (*n* = 4). **h** Gene expression of the target genes in STC-1 cells treated with 300 μM TβMCA for 12 h (*n* = 4). **i** Quantitation of the extracellular acidification rate (ECAR) by the seahorse assay. STC-1 cells were incubated with DMSO or TβMCA for 24 h and then successively injected with glucose (10 mM), oligomycin (1 μM), 2-deoxyglucose (100 mM) and rotenone (1 μM)/antimycin A (1 μM) for the seahorse flux assay at the indicated time. **j**–**l** Eight-week-old male WT or intestinal specific FXR KO (FXR^−/−^) mice were fed a high-fat diet for 5 weeks, and then treated with or without GP2-200 mg/kg/d for 2 weeks (*n* = 5–6). Intestinal *Fxr* mRNA levels (**j**) and pro-glucagon protein levels (**k**, **l**) from WT mice and FXR^−/−^ mice after GP2 treatment were detected. The results are shown as the mean ± s.e.m., *P < 0.05, **P < 0.01, ***P < 0.001 compared with vehicle or DMSO.

FXR activation in L cells inhibits pro-glucagon expression and GLP-1 secretion. Next, we asked whether oral administration of GP2 inhibited intestinal FXR transcription activity. As predicted, the gene expression of *Fxr* and its target genes small heterodimer partner (*Shp*) and fibroblast growth factor 15 (*Fgf15*) were much less in the ileum but not in the liver after GP2 administration (Fig. 3a). De-activation of intestinal FXR provides feedback to the liver and downregulates the transcriptional expression of *Cyp7a1* in the liver. Compared with vehicle treatment, GP2 treatment increased hepatic *Cyp7a1* and *Cyp8b1* gene expression (Fig. 3b). Intestinal FXR modulates the expression of genes associated with ceramide synthesis^20^. Consistently, oral administration of GP2 downregulated the mRNA levels of the ceramide synthesis-related genes *Cers5* and *Smptlc1* in the intestine compared with that in the vehicle-treated mice (Fig. 3c). These data suggested that intestinal FXR signalling was markedly decreased after GP2 treatment. Considering that up to 100 μM GP2 exhibited no obvious inhibitory effect in the in vitro FXR binding assay (Figure S6), these results further indicate that oral administration of GP2 indirectly inhibits the intestinal FXR signalling pathway.

### GP2 treatment inhibits BSH activity and increases intestinal TβMCA accumulation

Bile acid metabolites are involved in FXR regulation. To further explore the mechanism of intestinal FXR inhibition by GP2, we examined the concentrations of several taurine-conjugated bile acids after GP2 treatment. Compared with vehicle treatment, oral administration of GP2 increased the concentrations of fecal Tα/βMCA, which are endogenous FXR antagonists. In contrast to the levels of Tα/βMCA, the concentration of taurodeoxycholic acid (TDCA), an endogenous FXR agonist, was much lower in GP2-treated mice, whereas the fecal concentrations of taurine chenodeoxycholic acid (TCDCA) and taurocholic acid (TCA) were comparable between the two groups of mice (Fig. 3d). Besides, G-protein coupled bile acid receptor 1 (GPBAR1), also named TGR5, increases GLP-1 secretion once activated by bile acids^29,30^. Then, we measured the changes in bile acids related to TGR5 activation after GP2 treatment. The concentrations of deoxycholic acid, cholic acid (CA) and chenodeoxycholic acid (CDCA) in feces were not changed, whereas the lithocholic acid content in the feces was much lower in GP2-treated mice (Figure S7). Taken together, these results indicate that oral administration of GP2 increases the accumulation of intestinal Tα/βMCA.

Tα/βMCA are synthesised in the liver and deconjugated into α/βMCA and taurine by BSH in the gut. To verify the effects of GP2 on BSH in vivo, we measured BSH activity in the caecum after GP2 treatment. As predicted, GP2 treatment markedly abolished fecal BSH activity (Fig. 3e). To further examine the inhibitory effect of GP2 on BSH, we incubated the feces isolated from HFD-fed mice with GP2 and TCDCA-d5 in vitro. GP2 treatment dose-dependently decreased the de-conjugation rate of TCDCA-d5 to CDCA-d5 (IC_50_ = 479.5 ± 16.4 μM, Fig. 3f). These studies demonstrate that GP2 acts as a potential BSH inhibitor.

FXR activation decreases pro-glucagon mRNA expression by inhibiting glycolysis, and TβMCA treatment may improve GLP-1 production. To test this hypothesis, we incubated STC-1 cells with TβMCA for 24 h, and measured the GLP-1 content. In accordance with previous studies, TβMCA treatment triggered a dose-dependent increase in GLP-1 secretion (Fig. 3g) and induced much more pro-glucagon mRNA expression in STC-1 cells (Fig. 3h). Meanwhile, the glycolytic capacity was much higher after TβMCA treatment (Fig. 3i).

To further assess the effects of the intestinal FXR pathway on the amelioration of GLP-1 production and glucose homoeostasis induced by GP2 treatment, we administrated HFD-fed WT and intestine-specific *Fxr* knockout (FXR^−/−^) mice with GP2 for 2 weeks. The intestinal mRNA levels of *Fxr* in FXR^−/−^ mice were much lower than that in WT mice (Fig. 3j). Consistent with previous reports, intestinal GLP-1 protein levels were much higher in FXR^−/−^ mice than in WT mice (Fig. 3k, l). However, GP2 treatment resulted in significantly higher pro-glucagon protein expression in WT mice, but not in FXR^−/−^ mice (Fig. 3k, l). Meanwhile, GP2 treatment reduced fasting blood glucose levels and improved oral glucose tolerance in WT mice, but not in FXR^−/−^ mice (Figure S8a–S8f). Taken together, these results support the role of FXR pathway in intestinal GLP-1 production induced by GP2 treatment.

### GP2 treatment modifies the intestinal microbiota composition

**Fig. 4:**
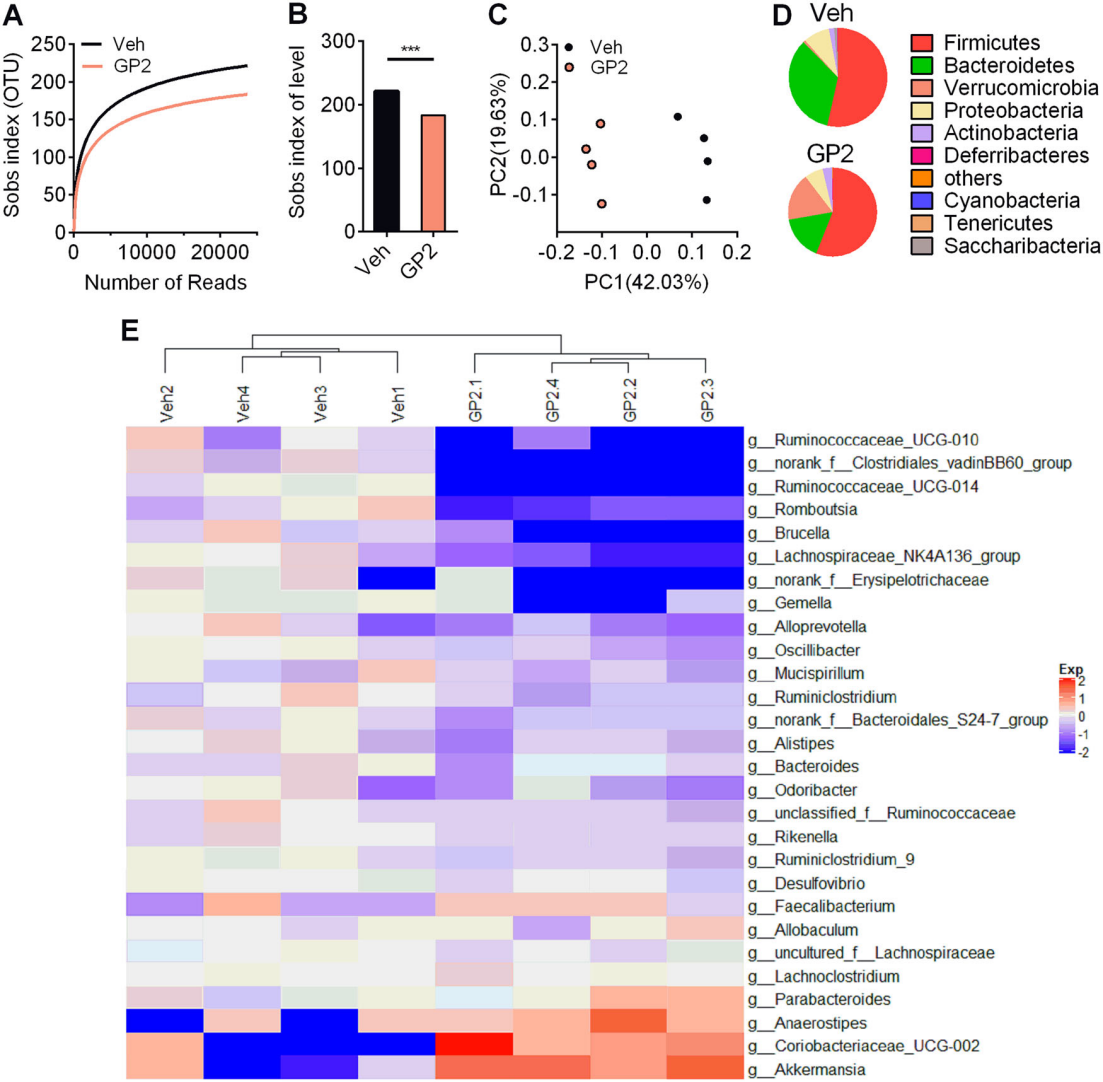
Oral administration of GP2 changes the gut microbiota composition. Six-week-old male mice were fed a high-fat diet for 8 weeks, and then treated with or without 200 mg/kg/d GP2 for 5 weeks (*n* = 4). **a**, **b** Sobs index analysis of the gut microbiome of HFD-fed mice after oral administration of GP2 for 5 weeks. **c** Principal coordinate analysis of gut microbiota composition based on unweighted UniFrac. **d** Pie charts of the average relative abundance of the gut microbiota in the feces at the phylum level. **e** Heatmap depicting the relative abundance of the microbiota in the feces at the genus level as in **d**. The results are shown as the mean ± s.e.m., ***P < 0.001 compared with vehicle.

To evaluate the modulation of the gut microbiota, we assessed microbial communities by 16 S rRNA sequencing. Compared with vehicle treatment, GP2 treatment decreased the microbiota phylogenetic diversity (Fig. 4a, b). PCoA showed a significant difference between GP2-treated mice and vehicle-treated mice (Fig. 4c). At the phylum level, oral administration of GP2 moderately decrease the proportion of *Bacteroidetes* and dramatically increased the proportion of *Verrucomicrobia* (Fig. 4d, Figure S9). Although the proportion of *Firmicutes* was similar between the two groups of mice, the genera *norank_f_Clostridiales_vadinBB60_group*, *Gemella*, *Ruminiclostridium_9*, *Romboutsia*, *Lachnospiraceae_NK4A136_group*, *Ruminococcaceae_UCG-014*, *Oscillibacter*, *Ruminococcaceae_UCG-010* and *Ruminiclostridium* from *Firmicutes* were less common in GP2-treated mice (Fig. 4e). GP2 treatment decreased the abundance of the genera *Brucella* and *Desulfovibrio* from *Proteobacteria* and the genera *norank_f_Bacteroidales_S24-7_group* from *Bacteroidetes* (Fig. 4e). The proportions of *Oscillibacter*, *Ruminiclostridium*, *Ruminiclostridium_9, norank_f_Clostridiales_vadinBB60_group*, *Desulfovibrio* and *norank_f_Bacteroidales_S24-7_group* but not *Lactobacillus*, which have been identified with highly BSH expression, were decreased after GP2 treatment (Fig. 4e, Figure S10). These results suggest that oral administration of GP2 modulates the gut microbiota, and this effect is probably associated with the inhibition of BSH enzymatic activity.

In addition, administration of GP2 increased the proportion of the phylum *Verrucomicrobia* (Fig. 4d) by increasing the abundance of genera *Akkermansia muciniphila* (*A. muciniphila*) (Figure S11a). *A. muciniphila* is a mucin-degrading bacterium^31^. In accordance with the obvious increase in *A. muciniphila*, the number of goblet cells, which are responsible for secreting mucins to protect the mucous membranes, were slightly increased in the intestine after GP2 treatment compared with after vehicle treatment (Figure S11b, S11c). In addition, oral administration of GP2 improved the permeability of the intestinal barrier (Figure S11d, S11e), and the expression of intestinal inflammation related genes were decreased after GP2 treatment (Figure S11f). Overall, these results further confirm that oral administration of GP2 improves microbiota homoeostasis in the gut of HFD-fed mice.

### GP2 treatment induces intestinal pro-glucagon expression via gut microbiota remodelling

**Fig. 5:**
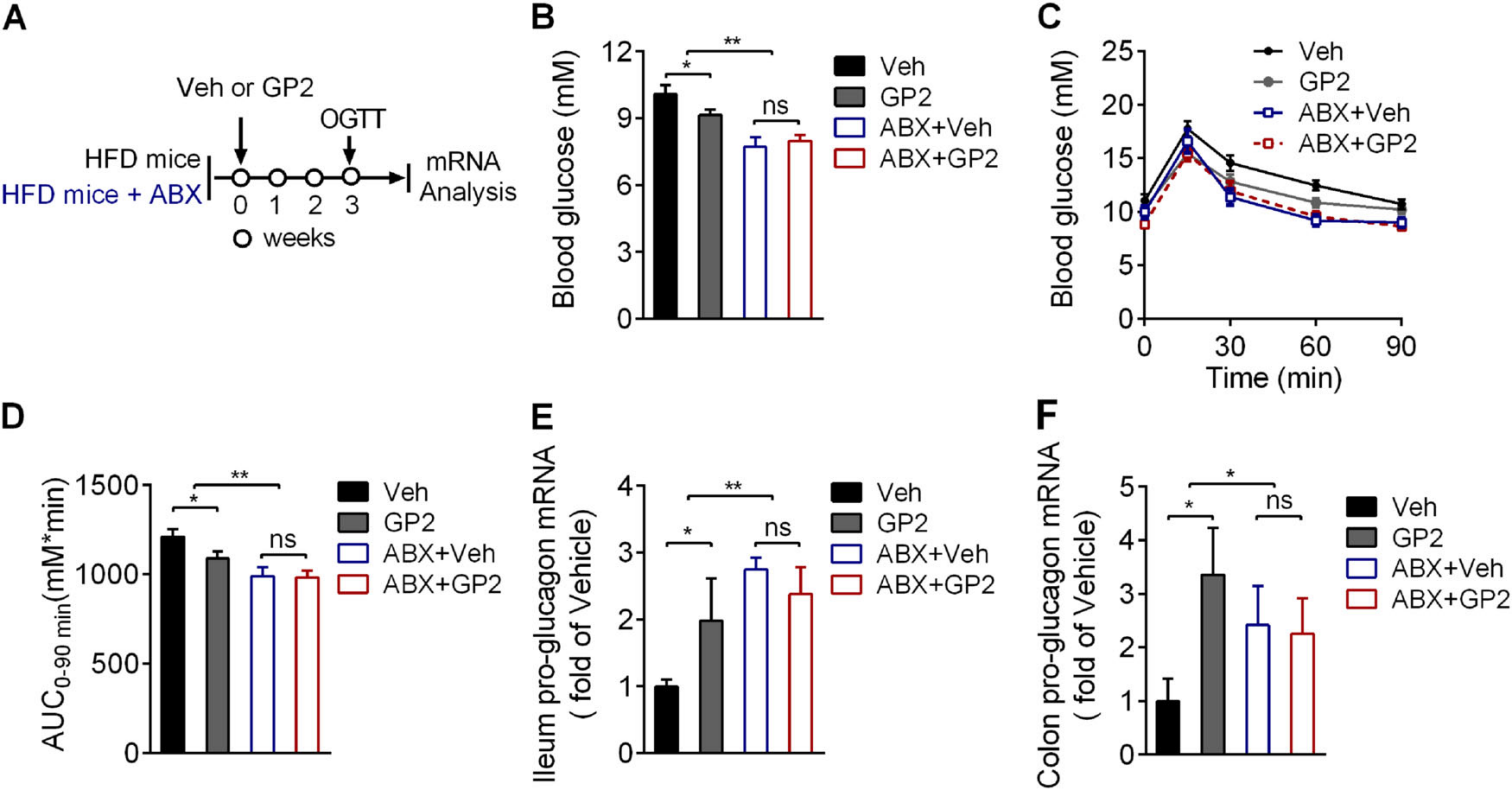
GP2 induces intestinal pro-glucagon expression through gut microbiota remodelling. **a** Schematic diagram of the experiment (*n* = 9–11). In brief, 6-week-old male mice were fed a high-fat diet for 10 weeks, and then the mice were randomly divided into four groups according to body weight and fasting blood glucose levels. Two groups of mice were treated with an antibiotic mix (ABX) for 1 week and then treated with or without GP2 combined with ABX for 3 weeks; the other two groups of mice were fed a high-fat diet for 1 week, and then treated with or without GP2 for 3 weeks. **b** Fasting blood glucose concentrations were measured after treatment with or without GP2 (200 mg/kg/d), and treatment with or without ABX for 3 weeks. The animals were fasted for 6 h before blood glucose determination. **c** The oral glucose tolerance test (OGTT) results of vehicle-, GP2-, vehicle+ ABX-and GP2+ABX-treated mice after glucose (2.0 g/kg) administration by gavage were plotted. **d** Area under the curve of glucose level in 90 min of OGTT in (**c**), **e**, **f** Quantitative mRNA expression of pro-glucagon in the ileum (**e**) and colon (**f**) after GP2 treatment combined with or without ABX treatment. The results are shown as the mean ± s.e.m., *P < 0.05, **P < 0.01, ***P < 0.001 compared with vehicle.

To confirm the role of the intestinal microbiota following GP2-induced FXR inhibition and GLP-1 secretion, we treated HFD-fed mice with an antibiotic cocktail (ABX) to confirm the role of the microbiota in promoting pro-glucagon gene expression and glucose-induced GLP-1 secretion after GP2 administration (Fig. 5a). Consistently, compared with vehicle, GP2 administration as well as ABX treatment alone decreased blood glucose levels and improved glucose tolerance in HFD-fed mice (Fig. 5b–d). However, the blood glucose lower effect and oral glucose tolerance improvement were not further induced by GP2 treatment in ABX treatment mice (Fig. 5b–d). Besides, oral administration of GP2 markedly induced pro-glucagon gene expression in the ileum and colon in the control mice but not further increased in ABX-treated mice (Figs. 5e, 5f). These results suggest that chronic GP2 treatment improves L cells function in a manner dependent on the presence of gut microbiota.

**Fig. 6:**
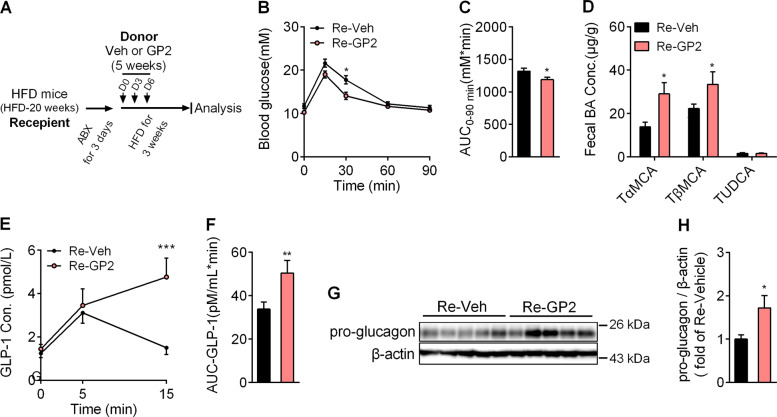
Transplantation of the fecal microbiota of GP2-treated mice increases GLP-1 secretion. **a** Schematic diagram of the pharmacodynamic experiment. In brief, 6-week-old male mice were fed a high-fat diet for 20 weeks, and then the gut microbiota was depleted by antibiotics in drinking water for 3 days. Twelve hours after last dose of antibiotics given, the mice were oral administered the feces (resuspended in PBS) isolated from vehicle (Re-Veh) or GP2-treated mice (Re-GP2) for three times (on day 0, day 3, day 6). Then the mice were fed a high-fat diet for another 3 weeks. **b** The mice were fasted for 6 h and an oral glucose tolerance test (OGTT) was conducted after glucose (2.0 g/kg) after administration by gavage (*n* = 8–10). **c** Area under the curve of glucose level in 90 min of OGTT in **b** (*n* = 8–10). **d** The levels of the individual taurine-conjugated bile acids TαMCA, TβMCA, and TUDCA were measured in the feces (*n* = 5–6). **e** Plasma concentration of active GLP-1 in Re-Veh and Re-GP2 mice in response to oral administration of 2.0 g/kg glucose (*n* = 5–6). **f** Area under the curve of active GLP-1 level in 15 min after glucose challenge in **e**. **g**, **h** Immunoblot (**g**) of pro-glucagon in RIPA lysis extracts from the ilea of Re-Veh and Re-GP2 mice and the quantification (**h**) (*n* = 5). The results are shown as the mean ± s.e.m., *P < 0.05, **P < 0.01, ***P < 0.001 compared with Re-Veh.

To further establish the role of microbiota in the maintenance of glucose homoeostasis, we transplated microbiota from GP2-treated or vehicle-treated mice by gavage into antibiotic-treated mice (Fig. 6a). As expected, mice transplanted with the gut microbiota from GP2-treated (Re-GP2) mice showed an improvement in glucose tolerance capacity (Fig. 6b, c). In addition, compared with Re-Veh mice, the fecal levels of Tα/βMCA were also markedly increased in Re-GP2 mice (Fig. 6d). Much more GLP-1 secretion was also induced after glucose challenge in Re-GP2 than in Re-Veh mice (Fig. 6e, f). Moreover, the pro-glucagon gene expression and pro-glucagon protein levels were higher in Re-GP2 mice than that in Re-Veh mice (Fig. 6g, h). Taken together, these data suggest that transplantation of the microbiota of GP2-treated HFD-fed mice is sufficient to induce pro-glucagon gene expression and glucose-induced GLP-1 secretion.

## Discussion

Gypenosides isolated from *G. pentaphyllum* have been identified to improve metabolic syndrome via insulin secretion stimulation, glucose uptake promotion and gut microbiota modulation^32–34^. In the present study, oral administration of GP2, a bioactive metabolite of gypenosides extracted from *G. yixingens*e, improved glucose homoeostasis in HFD-fed mice via the intestinal FXR/GLP-1 axis and these effects were dependent on gut microbiota remodelling.

Based on these findings, our data showed that GP2 treatment downregulated the enzymatic activity of fecal BSH in vitro and in vivo, which resulted in an increase in the contents of Tα/βMCA in the feces of GP2-treated mice. Consistent with the 16 S rRNA results, the accumulation of fecal TβMCA in GP2-treated mice and Re-GP2 mice was much higher than that in vehicle-treated mice and Re-Veh mice. These results indicate that GP2-mediated modulation of TβMCA metabolism is partly dependent on gut microbiota modulation. Previous studies indicated that FXR inhibition in intestinal epithelial cells downregulates ceramide synthesis in vivo^22,35,36^. Although GP2 administration lowered ceramide biosynthesis-related gene expression (*Cers5* and *Sptlc1*), the difference in the composition or content of ceramides between vehicle-treated mice and GP2-treated mice needs to be further explored.

Conjugated bile acids play a critical role in lipid solubilisation and are the target substrates of BSH. The inhibitory effect on microbiota growth induced by BSH inhibition may be associated with an increase in the bile acid concentration in the feces^37,38^. However, the latest published data showed that a covalent inhibitor of BSH does not inhibit the growth of the bacteria^11^. In our study, GP2 treatment decreased the phylogenetic diversity of the microbiota, especially the proportion of several genera that express high levels of BSH. The pattern of GP2-mediated BSH inhibition is unknown, and may be associated with a mild gut microbiota modulatory effect. Therefore, determining whether GP2 inhibits the growth of bacteria that express high levels of BSH gene requires additional analyses.

In addition, in agreement with a recent study^34^, we found that the beneficial effect of GP2 treatment on metabolic phenotypes was associated with a dramatic increase in the relative abundance of *A. muciniphila*. Oral administration of GP2 increased the abundance of *A. muciniphila* by 20-fold in HFD-fed mice (from 0.74% to 17.32%). These results indicate that gypenosides can increase the growth of *A. muciniphila* through an unknown mechanism. Although the contents of TβMCA in the Re-GP2 mice was much higher than that in the Re-Veh mice, the proportion of *A. muciniphila* was similar in Re-Veh mice and Re-GP2 mice (data not shown), which further demonstrate that GP2 treatment improves GLP-1 production and secretion in a manner dependent on intestinal FXR inhibition.

Finally, chronic administration of GP2 improved intestinal L-cell function, which was associated with the de-activation of intestine-specific FXR signalling pathway. These findings demonstrate that GP2 has therapeutic potential and may serve as an oral agent for metabolic syndrome.

## Supplementary information

Figure S1

Figure S2

Figure S3

Figure S4

Figure S5

Figure S6

Figure S7

Figure S8

Figure S9

Figure S10

Figure S11

Table S1

Table S2

Supplementary Materials and Methods

Supplementary Figure Legends
