# Supplementary material for: The triterpenoid sapogenin (2α-OH-Protopanoxadiol) ameliorates metabolic syndrome via the intestinal FXR/GLP-1 axis through gut microbiota remodelling
Source: Cell Death Dis. 2020 Sep 17;11(9):770. doi: 10.1038/s41419-020-02974-0 (PMC7499306; doi:10.1038/s41419-020-02974-0)
Supplement: Supplementary file 12 — Table S1 [file 41419_2020_2974_MOESM12_ESM.docx]

**Supplement Table**

| Table S1. Sequences of primer pairs used for RT-qPCR in this study | | |
| --- | --- | --- |
| Gene | Forward primer | Reverse primer |
| *Gapdh* | TGGTCCAGGGTTTCTTACT | TCTCCTGCGACTTCAACA |
| *Gcg* | GATCATTCCCAGCTTCCCAG | CTGGTAAAGGTCCCTTCAGC |
| *Fgf15* | ACGGCAAGATATACGGGCTG | GGCTTGGCCTGGATGAAGAT |
| *Fxr* | GCACGCTGATCAGACAGCTA | CAGGAGGGTCTGTTGGTCTG |
| *Shp* | CAGCGCTGCCTGGAGTCT | AGGATCGTGCCCTTCAGGTA |
| *Vdr* | GAAGCGCAAGGCCCTGTT | CGCTGCACCTCCTCATCTGT |
| *Pxr* | TAGGGACCTGCCTATTGAGGA | TAGGGACCTGCCTATTGAGGA |
| *Cyp7a1* | AACAACCTGCCAGTACTAGATAGC | GTGTAGAGTGAAGTCCTCCTTAGC |
| *Cyp7b1* | AATTGGACAGCTTGGTCTGCCT | TGTGTATGAGTGGAGGAAAGAGGG |
| *Cyp8b1* | GGCTGGCTTCCTGAGCTTATT | ACTTCCTGAACAGCTCATCGG |
| *Cyp27a1* | GCCTCACCTATGGGATCTTCA | TCAAAGCCTGACGCAGATG |
| *Sptlc1* | CGAGGGTTCTATGGCACATT | GGTGGAGAAGCCATACGAGT |
| *Cers2* | AAGTGGGAAACGGAGTAGCG | ACAGGCAGCCATAGTCGTTC |
| *Cers4* | GGATTAGCTGATCTCCGCAC | CCAGTATGTCTCCTGCCACA |
| *Cers5* | GTGTCATTGGGTTCCACCTT | CTTCTCCGTGAGGATGCTGT |
| *Smpd1* | GTTACCAGCTGATGCCCTTC | AGCAGGATCTGTGGAGTTG |
| *Smpd3* | CCTGACCAGTGCCATTCTTT | AGAAACCCGGTCCTCGTACT |
| *Smpd4* | ACCTGGCCCTCAATCCATTTG | ATAGGCACAGTCCGAAGTACG |
| *Erm1* | ATGGACAAACCAACTTTCAAGGC | GCAGACTGAGTTAGGACCACAA |
| *CD68* | CCATCCTTCACGATGACACCT | GGCAGGGTTATGAGTGACAGTT |
| *TNF-α* | CTGAACTTCGGGGTGATCGG | GGCTTGTCACTCGAATTTTGAGA |
