# Supplementary material for: The triterpenoid sapogenin (2α-OH-Protopanoxadiol) ameliorates metabolic syndrome via the intestinal FXR/GLP-1 axis through gut microbiota remodelling
Source: Cell Death Dis. 2020 Sep 17;11(9):770. doi: 10.1038/s41419-020-02974-0 (PMC7499306; doi:10.1038/s41419-020-02974-0)
Supplement: Supplementary file 13 — Table S2 [file 41419_2020_2974_MOESM13_ESM.docx]

**Supplement Table**

**Table S2 related to Figure 1**

| Table S2. Metabolic stability of GP2 in liver microsomes | | |
| --- | --- | --- |
| Product Name | Metabolic stability | |
|  | Species | MF % |
| GP2 | Human | 2 |
|  | Mouse | 0 |
|  | Rat | 14 |
