# Supplementary material for: The triterpenoid sapogenin (2α-OH-Protopanoxadiol) ameliorates metabolic syndrome via the intestinal FXR/GLP-1 axis through gut microbiota remodelling
Source: Cell Death Dis. 2020 Sep 17;11(9):770. doi: 10.1038/s41419-020-02974-0 (PMC7499306; doi:10.1038/s41419-020-02974-0)
Supplement: Supplementary file 14 — Supplementary Materials and Methods [file 41419_2020_2974_MOESM14_ESM.doc]

**1. Supplementary Materials and Methods**

**1.1 Chemicals and reagents**

Gypenosides (GPT) and GP2 were prepared as previously reported ^1,2^. INT-777, vancomycin, ampicillin, metronidazole and neomycin were purchased from MedChem Express (New Jersey, USA). TβMCA was purchased from Toronto Research Chemicals (Toronto, Canada). d5-TCDCA was purchased from ZZSRM (Shanghai, China). DMSO and Kolliphor EL were purchased from Sigma-Aldrich (Missouri, USA). GLP-1 antibody (ab23468, RRID: AB_470325) was purchased from Abcam; β-actin antibody (AM1021B, RRID: AB_2257945) was purchased from Abgent; Akt (4685S, RRID: AB_2225340), pS473-Akt (4051S, RRID: AB_331158), pT308-Akt (13038s, RRID: AB_2629447) antibodies were purchased from Cell Signaling Technology (Massachusetts, USA); IR (sc-711, RRID: AB_631835) and pT1162/1163-IR (sc-25103-R, RRID: AB_669389) antibodies were purchased from Santa Cruz (Dallas, USA); UCP-1 antibody (UCP11-A, RRID: AB_1624298) was purchased from Alpha Diagnostic (New Jersey, USA); α-tubulin antibody (T9026, RRID: AB_477593) was purchased from Sigma-Aldrich. A high fat diet (60 kcal %， D12492) was purchased from Research Diets Inc. (New Jersey, USA).

**1.2 Pharmacokinetic study**

Male *ob/ob* mice, HFD-induced mice, C57 mice or ICR mice were orally administered a single dose of 544 mg/kg GPT or 200 mg/kg GP2 after overnight fasting. Blood samples and tissues were collected at the indicated times, plasma and tissues were harvested and stored at -80°C prior to analysis. The concentration of GP2 was detected by HPLC-MS/MS^3^.

**1.3 Metabolic Animal Experiments**

For the glucose tolerance test, mice were fasted for 6 h and then given a single oral administration of glucose (2.0 g/kg). Tail vein blood was collected at the indicated time after glucose injection, and blood glucose was measured with an *ACCU-Chek* *performa* glucose meter (Roche, Basel, Switzerland). For the insulin tolerance test, mice were fasted for 4 h and intraperitoneally injected with recombinant human insulin (0.75 U/kg), and blood glucose was measured over the same time course as for the OGTT.

For the glucose stimulated GLP-1 and insulin secretion tests, mice were fasted for 6 h and then given a single oral administration of glucose (2.0 g/kg). Blood samples were collected from the orbital venous plexus before injection (0 min), 5 min and 15 min post-injection. Plasma was measured with ELISA kits for active GLP-1 (EGLP-35K, Millipore, Massachusetts, USA) and insulin (EZRMI-13K, Millipore, Massachusetts, USA).

For cold exposure experiments, singly-housed male mice were placed in pre-chilled cages and exposed to a 4 ℃ environment for the indicated time periods.

For depletion of the gut microbiota, fresh antibiotics containing 0.5 g/L neomycin, 0.5 g/L vancomycin, 1 g/L metronidazole, 1 g/L ampicillin were administered for 3 days in drinking water as described previously^4^. For faecal microbiota transplantation, the contents of the cecum from GP2-treated or vehicle mice were suspended in PBS, and 200 μL was administered orally to each male antibiotic-treated mouse every three days for a total of three times. The OGTT was performed after 2 weeks of faecal microbiota transplantation, and glucose-stimulated GLP-1 secretion was performed 3 weeks after faecal microbiota transplantation.

At the end of the experiment, the mice were fasted for 6 h and then euthanized, the livers and adipose tissues were collected and weighed. Some of the tissues were frozen in liquid nitrogen immediately for molecular and biochemical measurements, and the other tissues were fixed in 4% paraformaldehyde for histological analysis. Plasma levels of triglyceride and cholesterol were measured using the triglyceride determination kit (1.02.1801, Shanghai Changzheng, Shanghai, China) and cholesterol determination kit (1.02.0401, Shanghai Changzheng, Shanghai, China) according to the manufacturer’s instructions. For hepatic triglyceride analysis, 20-30 mg of livers were homogenized in PBS and mixed with CH_3_OH-CHCl_3_ (1:2, v/v). Then, the organic phase was transferred, air-dried overnight, and re-suspended in 1% Triton X-100 in absolute ethanol. The concentration of triglyceride was determined using the plasma triglyceride determination kit.

Whole-body fat and lean mass were measured by ^1^H-NMR spectroscopy (Minispec LF90 II, Bruker, Karlsruhe, Germany). Oxygen consumption (VO_2_), carbon dioxide (VCO_2_) production and locomotor activity were measured by using a sixteen-chamber indirect calorimeter (TSE PhenoMaster, TSE system, Germany) according to the manufacturer’s instructions. Heat production and RER (VCO_2_/VO_2_ ratio) were calculated as described previously.

**1.4 RNA extraction and quantification**

Total RNA was isolated from cells or tissues with TRIzol reagent (Invitrogen, California, USA). cDNA was synthesized with PrimeScript Reverse Transcriptase (TaKaRa, Japan) according to the manufacturer’s instructions. Real-time quantitative PCR was performed with SYBR Green PCR Master Mix (B21703, Bimake, China) on a Stratagene Mx3005P system (Agilent Technologies). The results were analysed by the ΔCt method and normalized to reference gene (β-actin or GAPDH). The sequences of the primer pairs used in the study are listed in Supplementary Table 1.

**1.5 Analysis of oxygen consumption and glycolytic rates**

STC-1 cells were seeded at a density of 1 x 10^4^ cells/well in 0.5% matrix gel coated XF96-well plates and allowed to grow for 24 h before treatment. The extracellular acidification rate (ECAR, mpH/min) was measured in real time at 37 °C using a Seahorse XF96 Extracellular Flux Analyzer instrument according to the manufacturer's instructions. For ECAR measurement, 10 mM glucose, 1 μM oligomycin, 100 mM 2-deoxyglucose and 1 μM rotenone plus 1 μM antimycin A were injected at different times.

**1.6 Western blot analysis**

Lysates of cells or tissues were prepared in RIPA lysis buffer (P0013B, Beyotime, Beijing, China) containing 1 mM Na_3_VO_4_, 10 mM NaF and complete protease inhibitor cocktail (04693116001, Roche, Basel, Switzerland) on ice and clarified by centrifugation at 12,000 g for 15 min at 4°C. Proteins were separated by SDS-PAGE after boiling for 10 min in loading buffer and transferred to NC membranes. The blots were blocked with 5% milk (in TBST) at room temperature for 1 h, incubated with the indicated primary antibodies overnight at 4°C, and incubated with secondary antibodies at room temperature for 1 h, and then developed with enhanced chemiluminescence substrate (RPN2232, GE Healthcare, USA). The protein bands were imaged with a ChemiDoc MP Imaging System (Bio-Rad, USA) and quantified with ImageJ Pro Plus.

**1.7 H&E staining and PAS staining**

Tissues were collected, washed or ﬂushed with PBS, immediately ﬁxed in 4% paraformaldehyde overnight at room temperature and then desiccated and embedded in parafﬁn. The specimens were cut into 4-6 μm sections and stained with haematoxylin and eosin (H&E) or periodic acid and Schiff (PAS) atain.

**1.8** **Bile acid analysis**

Faeces were prepared by precipitation and then mixed with dd water for 12 h at 4 ℃. An equal volume of tert-butanol was added, and samples were extracted for 45 min at 37 ℃. The supernatant, which contained bile acids, was transferred after centrifugation at 2,000 g for 15 min. The bile acid concentrations in the supernatants were measured by LC-MS/MS. Chromatographic separation was performed on a Thermo C18 column (2.1×100 mm, 5.0 μm) with a flow rate of 0.2 ml/min. A mobile phase consisting of water containing 0.1% formic acid and 5 mM ammonium acetate (A) and methanol (B) was used for gradient elution with the following sequence: 0→3.0 min: 35% B; 3.1 min→10.0 min: 45% B; 10.1 min→21.0 min: 50% B→80% B; 21.1 min→23.4 min: 80%→90% B; 23.4 min→26.0 min: 90％ B; and 26.1 min→29.0 min: 35% B^5,6^.

2. **Supplementary References**

1 Hu, L., Chen, Z. & Xie, Y. New triterpenoid saponins from Gynostemma pentaphyllum. *J Nat Prod* **59**, 1143-1145 (1996).

2 Dong, C. *et al.* Discovery, synthesis, and structure-activity relationships of 20S-dammar-24-en-2alpha,3beta,12beta,20-tetrol (GP) derivatives as a new class of AMPKalpha2beta1gamma1 activators. *Bioorg Med Chem* **24**, 2688-2696 (2016).

3 Zhang, L. *et al.* Simultaneous determination of gypenoside LVI, gypenoside XLVI, 2alpha-OH-protopanaxadiol and their two metabolites in rat plasma by LC-MS/MS and its application to pharmacokinetic studies. *J Chromatogr B Analyt Technol Biomed Life Sci* **1005**, 9-16 (2015).

4 Caesar, R. *et al.* Crosstalk between Gut Microbiota and Dietary Lipids Aggravates WAT Inflammation through TLR Signaling. *Cell Metab* **22**, 658-668 (2015).

5 Yang, L. *et al.* Bile acids metabonomic study on the CCl4- and alpha-naphthylisothiocyanate-induced animal models: quantitative analysis of 22 bile acids by ultraperformance liquid chromatography-mass spectrometry. *Chem Res Toxicol* **21**, 2280-2288 (2008).

6 Sun, L. *et al.* Gut microbiota and intestinal FXR mediate the clinical benefits of metformin. *Nat Med* **24**, 1919-1929 (2018).
