# Supplementary material for: The triterpenoid sapogenin (2α-OH-Protopanoxadiol) ameliorates metabolic syndrome via the intestinal FXR/GLP-1 axis through gut microbiota remodelling
Source: Cell Death Dis. 2020 Sep 17;11(9):770. doi: 10.1038/s41419-020-02974-0 (PMC7499306; doi:10.1038/s41419-020-02974-0)
Supplement: Supplementary file 15 — Supplementary Figure Legends [file 41419_2020_2974_MOESM15_ESM.docx]

**Supplement Figure**

**1.1 Supplementary Figure 1 related to Figure 1**

**Supplementary Figure 1. GP2 is a metabolite *in vivo* of Gy1 and Gy2. A**: The structures of Gy1, Gy2 and GP2. **B**: The concentration of GP2 was measured in the ileum 1 h after oral of a single dose of GPT (544 mg/kg, n=3-4) in the NC-mice, HFD-mice and *ob/ob* mice. The results are shown as the mean ± s.e.m., *P<0.05, **P<0.01, ***P<0.001 compared to C57 mice.

**1.2 Supplementary Figure 2 related to Figure 1**

**Supplementary Figure 2. GP2-treated mice are resistant to obesity-associated metabolic disorders.** **A, B**: Plasma ALT (A), AST (B) levels in HFD-mice treated with or without GP2-200 mg/kg (n=8-10). **C-F**: Immunoblot (C) of insulin receptor and AKT in RIPA lysis extracts from liver and quantification (D-F). Briefly, mice were fasted for 6 h and treated with insulin (5 U/kg for 5 min) via inferior vena, total liver lysates were immunoprecipitated using indicated antibodies. **G:** Daily food intake during treatment (n=8-10). **H**: Fat content was measured in the feces. **I**-**L**: CO_2_ production (I, J) and physical activity (K, L) were monitored over a 24 h period after 4 weeks of GP2 treatment. The results are shown as the mean ± s.e.m., *P<0.05, **P<0.01, ***P<0.001 compared to vehicle.

**1.3 Supplementary Figure 3 related to Figure 1:**

**Supplementary Figure 3. Chronic oral administration of GP2 improves thermogenesis in HFD-mice. A**: Core temperatures of vehicle or GP2-treated mice during cold exposure (4℃) at the indicated time. **B**: Relative mRNA levels of indicated genes in brown adipose tissue of the HFD-mice treated with vehicle or GP2 for 5 weeks (n=7-8). **C**: Immunoblots (left panel) and quantification (right panel) of UCP-1 in RIPA lysis extracts from brown adipose tissue, the relative optical density of UCP-1/α-tubulin were determined (n=6). The results are shown as the mean ± s.e.m., *P<0.05, **P<0.01, ***P<0.001 compared to vehicle.

**1.4 Supplementary Figure 4 related to Figure 2:**

**Supplementary Figure 4.** The concentration of GP2 in the duodenum, ileum, colon, liver (A) and plasma (B) after a single oral of GP2 (200 mg/kg) at the indicated time in the normal chow 8-week-old ICR mice.

**1.5 Supplementary Figure 5 related to Figure 2**

**Supplementary Figure 5. Administration of GP2 has no effect on Dpp-4 enzymatic activity *in vivo*.** Dpp-4 activity in plasma at the indicated time of 8-week-old male normal chow ICR mice after a single oral of 200 mg/kg GP2 or vehicle (n =7-8). Dpp-4 inhibitor alogliptin (3 mg/kg) acted as a positive control. The data represents mean ± s.e.m.. ***P<0.001 compared to vehicle at the same time point.

**1.6 Supplementary Figure 6 related to Figure 3**

**Supplementary Figure 6. GP2 does not inhibit FXR activity *in vitro*.** FXR activity was detected with a luciferase reporter according to the protocol (luciferase assay system, promega). Briefly, LBD (hFXR)-pbind and Luciferase reporter plasmids were co-transfected into HEK293 cell line for 12 h, and GP2 was added to the culture medium. 30 min later, 10 μM GW4064 was added to the culture medium, GP2 and GW4064 were co-incubated for another 24 hours. Luciferase assay were detected with Envision 2104. Data represents mean ± s.e.m.

**1.7 Supplementary Figure 7 related to Figure 3**

**Supplementary Figure 7. Individual bile acids level in the faeces after GP2-200 mg /kg treatment for 2 weeks in HFD-mice.** The bile acid profile in the faeces were plotted. LCA, lithocholic acid; DCA, ursodeoxycholic acid; CA, cholic acid; CDCA, chenodeoxycholic acid. Data represents mean ± s.e.m.. *P<0.05 compared to vehicle.

**1.8 Supplementary Figure 8 related to Figure 3**

**Supplementary Figure 8. GP2 treatment improves glucose homeostasis through the intestinal FXR signaling pathway. A, B**: Mice were fasted for 6 h, and the fasting blood glucose levels of WT mice (A) and FXR^-/-^ mice (B) were measured after GP2 treatment for 2 weeks. **C-F**: WT mice and FXR^-/-^ mice were subjected to oral glucose tolerance test (2.0 g/kg glucose) on day 12 of treatment. The area under the curve of glucose level in 120 minutes of OGTT. The results are shown as the mean ± s.e.m., *P<0.05, **P<0.01, ***P<0.001 compared to control mice.

**1.9 Supplementary Figure 9 related to Figure 4**

**Supplementary Figure 9. Comparison of phylum-level proportional abundance in the faeces of GP2-treated mice and vehicle-treated mice.**

**1.10 Supplementary Figure 10 related to Figure 4:**

**Supplementary Figure 10**. 16s rRNA gene sequencing analysis of genera *Lactobacillus* in the faeces of vehicle-treated and GP2-treated mice (n=4). Richness was represented as the proportions of OTUs and was normalized to vehicle.

**1.11 Supplementary Figure 11 related to Figure 4**

**Supplementary Figure 11. HFD-mice treated with GP2 shows a higher abundance the genera *Akkermansia*.** **A**: 16s rRNA gene sequencing analysis of genera *Akkermansia* in the faeces of vehicle-treated and GP2-treated mice (n=4). Richness was represented as the proportions of OTUs and was normalized to vehicle. **B**, **C**: Goblet cells in the ileum of vehicle-treated and GP2-treated mice were indicated by PAS-Ab staining (n=5), the number of goblet cells was quantified with Image J Pro Plus. **D**: The concentration of FITC dextran 4000 in the plasma were detected in the vehicle-treated and GP2-treated mice. The mice were fasted for 6 h and oral administration of a single dose of 500 mg/kg FITC dextran 4000, the concentration of FITC dextran 4000 was measured at the indicated time (n=8). **E**: Area under the curve of FITC dextran 4000 level in D. **F**: The mRNA expression of the target genes in the ileum were measured after GP2 treatment for 5 weeks. The results are shown as the mean ± s.e.m., *P<0.05, **P<0.01 compared to control mice.
